# Supplementary material for: Dams threaten salmonids by triggering temperature-dependent proliferative kidney disease
Source: Commun Biol. 2026 Jan 9;9:192. doi: 10.1038/s42003-025-09470-1 (PMC12881449; doi:10.1038/s42003-025-09470-1)
Supplement: Supplementary file 2 — Supplementary Material [file 42003_2025_9470_MOESM2_ESM.pdf]

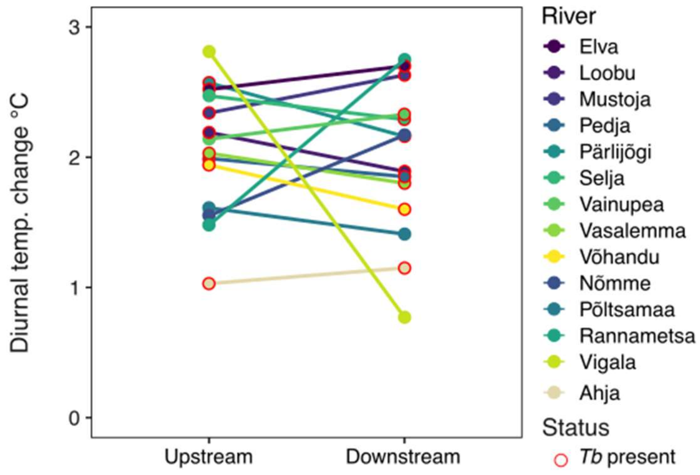

Supplementary Figure 1: Diurnal water temperature changes up- vs. downstream of the dams.

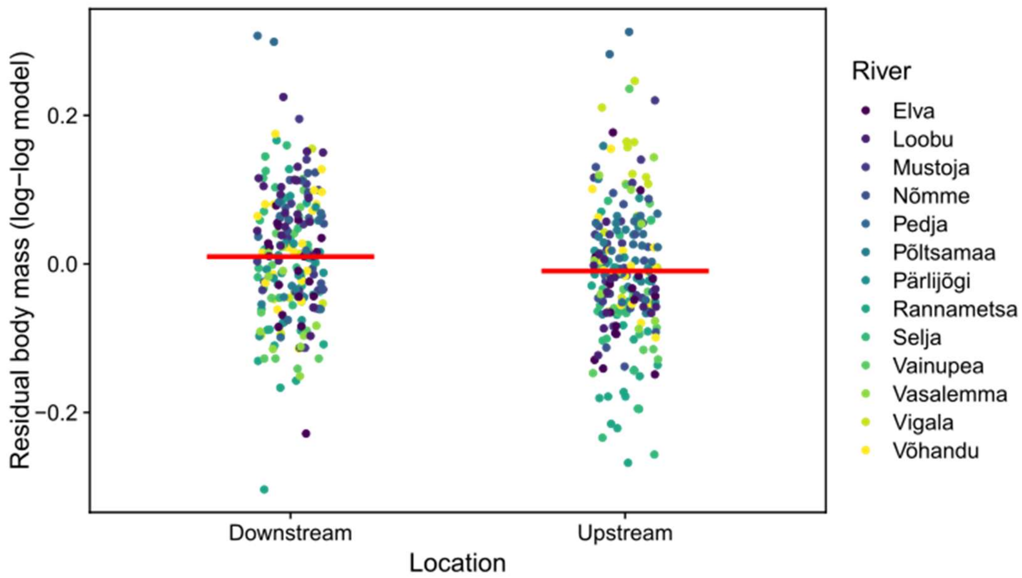

Supplementary Figure 2: Residual body mass for downstream and upstream locations. Residuals from model: $\log_e(m) \sim \log_e(tl) + location + (1|river)$. Red horizontal line shows the overall mean.

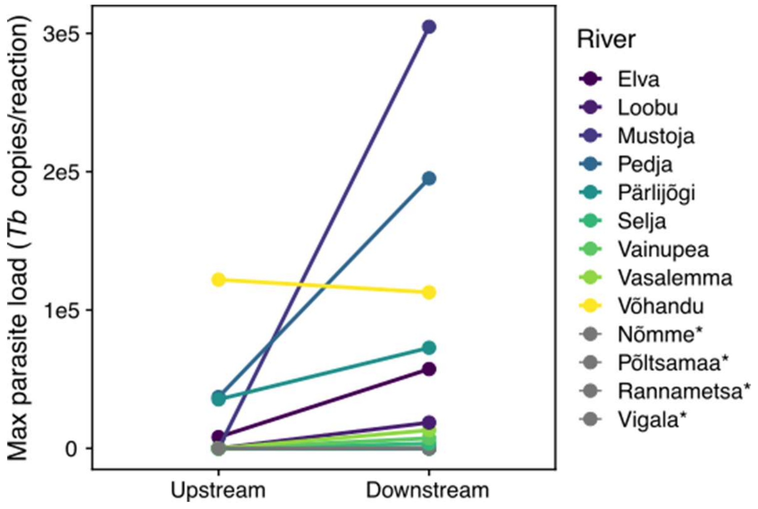

Supplementary Figure 3: Maximum parasite load (*Tb* copies/reaction) up- and downstream of dams (asterisk denotes rivers without *Tb*).

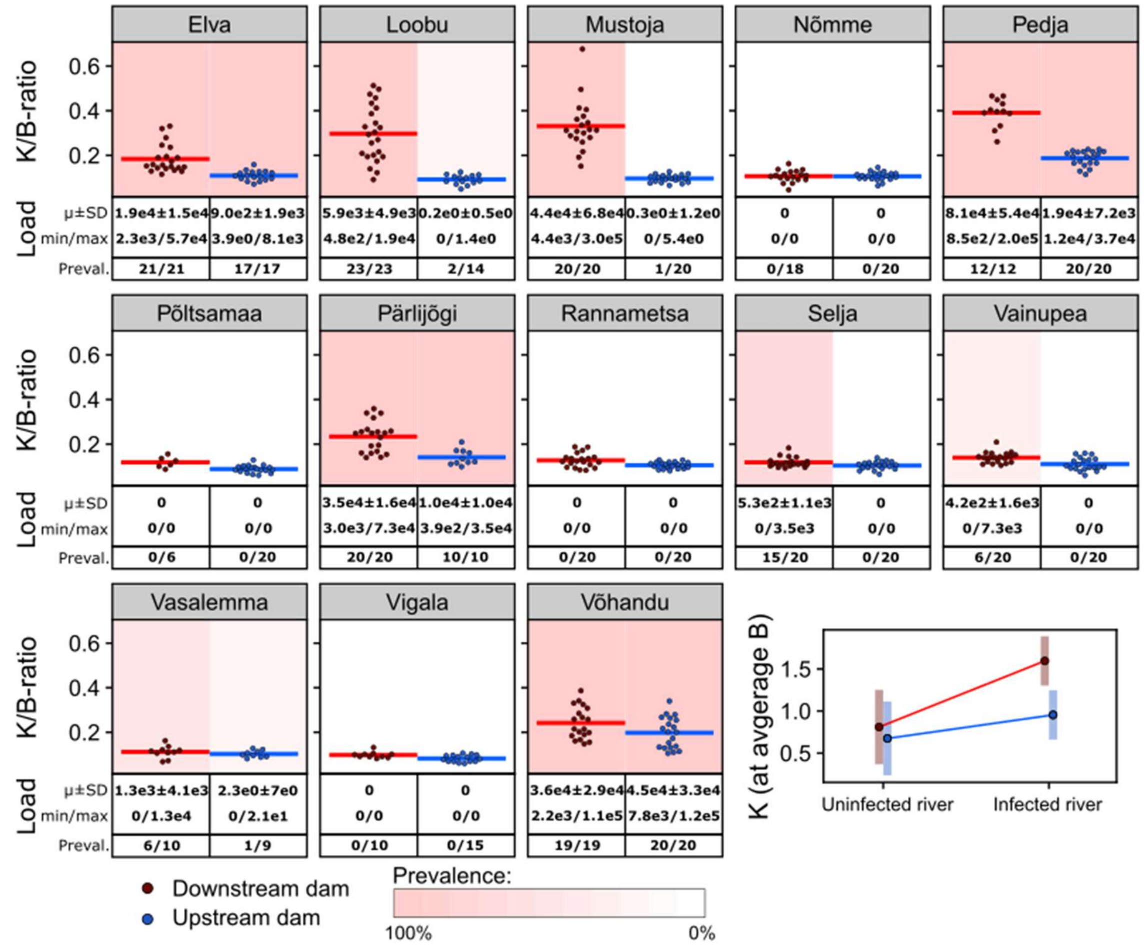

Supplementary Figure 4: Summarizing figure of renal hyperplasia (K/B-ratio; kidney thickness divided by dorsal muscle thickness), parasite loads (Load; *Tb* copies/reaction) and prevalence (% infected individuals) in brown trout in Estonian rivers (upstream and downstream of dams). Parasite loads are summarised for each river, in terms of mean and standard deviation (μ ± SD), minimum and maximum values (min/max), and number of individuals with detected loads out of total sample ('Preval.'). For each river location, the prevalence is also indicated by graph background shading. The K/B-ratio is presented for each river (points: individual fish; horizontal bar = mean), as well as in the form of estimated marginal means with 95% confidence intervals (lower right); in the latter graph, the estimates relate to the mean kidney thickness (mm) at the mean body thickness.

Supplementary Table 1: Sampling site information with dam/reservoir characteristics, parasite prevalence and temperature estimates.

River - Dam name	Date (dd.mm.yyyy)	Location from dam	Coordinates (N, E)	Distance from the dam (km)	No. of juveniles sampled	No. of infected	No. of uninfected	*Tb* prev. (95% CI)	Parasite load (*Tb* copies/reaction)	K/B ratio	Hematocrit	Dam height (m)	Reservoir size (ha)	Average water temp. (°C)	No. Of days > 15 °C	Diurnal water temp. difference (°C)
Rannametsa - Laiksaare	22.08.2022	Downstream	58°05' 46.5, 24°40' 02.7"	0.1	20	0	20	0 (0−0.161)	0	0.127	0.329	3.35	1.2	19.68	55	2.75
Rannametsa – Laiksaare	22.08.2022	Upstream	58°05' 40.3, 24°40' 23.6"	0.4	20	0	20	0 (0−0.161)	0	0.105	0.336			16.45	45	1.48
Vigala – Kuusiku	23.08.2022	Downstream	58°57' 54.3, 24°43' 01.4"	0.1	10	0	10	0 (0−0.277)	0	0.098	0.368	2.15	5.4	17.51	55	0.77
Vigala – Kuusiku	23.08.2022	Upstream	58°55' 25.7, 24°51' 09.0"	11.8	15	0	15	0 (0−0.204)	0	0.082	0.299			12.20	2	2.81
Vainupea – Pajuveski	24.08.2022	Downstream	59°34' 02.4, 26°15' 28.7"	0.2	20	6	14	0.2 (0.145−052)	1404.7	0.139	0.321	2.65	0.6	19.47	55	2.33

Vainupea – Pajuveski	24.08.2022	Upstream	59°34' 02.4, 26°15' 28.7"	1.8	20	0	20	0 (0−0.161)	0	0.110	0.341			17.50	53	2.14
Selja - Päide	25.08.2022	Downstream	59°23' 27.4", 26°23' 30.4"	7.7	20	15	5	0.75 (0.531−0.888)	699.5	0.118	0.379	2.25	3.1	18.29	55	2.29
Selja - Päide	25.08.2022	Upstream	59°22' 31.5, 26°17' 13.6"	2.9	20	0	20	0 (0−0.204)	0	0.104	0.333			14.14	14	2.47
Mustoja - Vihula II/III	25.08.2022	Downstream	59°33' 09.9, 26°10' 59.3"	1.5	20	100	0	1 (0.839−1)	44255	0.331	0.29	2.55/6	2.2/10.6	15.72	55	2.63
Mustoja - Vihula II/III	26.08.2022	Upstream	59°31' 48.0, 26°10' 44.4"	2	20	1	19	0.05 (0−0.236)	5.4	0.096	0.382			13.38	48	2.34
Põltsamaa – Ao	27.08.2022	Downstream	59°00' 22.7, 26°12' 27.7"	0.1	6	0	6	0 (0−0.39)	0	0.118	0.318	0.95	10.3	19.24	55	1.41
Põltsamaa – Ao	27.08.2022	Upstream	59°03' 30.8, 26°10' 38.8"	9.9	20	0	20	0 (0−0.161)	0	0.088	0.359			13.50	4	1.61
Nõmme -	28.08.2022	Downstream	59°02' 32.8,	0.2	18	0	18	0 (0−0.176)	0	0.107	0.35	3.2	5.7	15.72	34	2.17

Nõmme veski			26°13' 29.7"													
Nõmme - Nõmme veski	28.08.2022	Upstream	59°02' 42.1, 26°14' 20.1"	1	20	0	20	0 (0−0.161)	0	0.106	0.313			13.38	7	1.55
Ahja – Saesaare	29.08.2022	Downstream	58°06' 52.2, 27°03' 05.5"	0.6	0	N/A	N/A	N/A	N/A	N/A	N/A	7.65	48.5	19.02	55	1.15
Ahja – Saesaare	29.08.2022	Upstream	58°08' 38.9, 26°58' 28.9"	12.6	15	15	0	1 (0.796−1)	10938.1	0.121	0.34			16.57	50	1.03
Võhandu – Hutita	31.08.2022	Downstream	57°53' 00.1, 26°44' 32.5"	0.1	19	19	0	1 (0.832−1)	35690	0.242	0.339	2.25	2.1	16.87	50	1.6
Võhandu – Hutita	29.08.2022	Upstream	57°55' 32.7, 26°45' 05.5"	7.4	20	20	0	1 (0.839−1)	44827.5	0.198	0.297			15.92	42	1.94
Pärlijõgi - Alaveski	31.08.2022	Downstream	57°45' 23.6, 26°45' 55.6"	4.3	20	20	0	1 (0.839−1)	35368.9	0.234	0.329	1	0.5	15.63	36	2.16
Pärlijõgi - Alaveski	31.08.2022	Upstream	59°02' 42.1, 26°14' 20.1"	1.1	10	10	0	1 (0.722−1)	10468	0.141	0.407			15.39	32	2.57

Elva - Hellenurme	01.09.2022	Downstream	58°08' 33.8, 26°23' 51.7"	1	21	21	0	1 (0.845−1)	19150.3	0.183	0.384	2.9	5.5	18.99	55	2.7
Elva - Hellenurme	01.09.2022	Upstream	58°06' 54.8, 26°23' 17.7"	3.8	17	17	0	1 (0.816−1)	897.9	0.109	0.368			16.10	41	2.52
Pedja – Käruveski	02.09.2022	Downstream	57°45' 23.6, 26°45' 55.6"	3.6	12	12	0	1 (0.757−1)	80640	0.19	0.391	1	1	17.45	53	1.85
Pedja – Käruveski	02.09.2022	Upstream	58°56' 52.8, 26°30' 25.4"	5.7	20	20	0	1 (0.839−1)	19100.2	0.187	0.36			15.35	32	1.99
Vasalemma – Töökmani	05.09.2022	Downstream	59°12' 25.1, 24°25' 06.7"	0.3	10	6	4	0.6 (0.313−0.832)	2224.1	0.112	0.443	1	0.6	17.19	51	1.8
Vasalemma – Töökmani	05.09.2022	Upstream	59°12' 10.4, 24°26' 10.6"	1.1	10	1	9	0.1 (0−0.04)	21	0.103	0.346			16.97	48	2.03
Loobu - Undla/Kadrina	30.08.2023	Downstream	59°21' 00.5, 26°06' 31.8"	0.2	23	23	0	1 (0.857−1)	5903.8	0.297	0.295	1/2.65	0.5/15.5	18.70	55	1.89
Loobu - Undla/Kadrina	30.08.2023	Upstream	59°18' 46.5,	2.5	15	2	13	0.13 (0−0.038)	1.3	0.092	0.377			16.35	47	2.19

	26°09' 53.4"

Supplementary Table 2: Summary table from linear mixed model for analysis of total length (mm): tl ~location + (1|river).

Random effects

Groups		Variance	SD		
River	(Intercept)	85.9	9.3		
Residual		106.9	10.3		

N (obs.): 442
N (River): 13

Fixed effects

	Estimate	SE	df	*t*	*P*
(Intercept)	74.2	2.7	13.1	27.8	5.17e-13
Location (upstream)	-0.16	1.00	429.4	-0.16	0.870

Supplementary Table 3: Summary table from linear mixed model for analysis of body condition (relative total mass, g): tm ~ log(tl) + location + (1|river).

Random effects

Groups		Variance	SD		
River	(Intercept)	0.002	0.042		
Residual		0.007	0.081		

N (obs.): 442
N (River): 13

Fixed effects

	Estimate	SE	df	*t*	*P*
(Intercept)	-12.32	0.12	371.2	-106.7	< 2e-16
$\log_e$(total length)	3.18	0.03	388.8	118.9	< 2e-16
Location (upstream)	-0.02	0.01	430.0	-2.83	0.005

Supplementary Table 4: Summary table from binomial generalized mixed model (logit-link) for analysis of parasite prevalence: PV ~ location + (1|river); PV is the proportion of *Tb*-positive samples and the total sample size for each river was used as weights in the model. Model run on all data.

Random effects

Groups		Variance	SD
River	(Intercept)	79.49	8.92

N (obs.): 26
N (River): 13

Fixed effects

	Estimate	SE	*z*	*P*
(Intercept)	2.01	2.99	0.67	0.502
Location (upstream)	-4.96	0.76	-6.50	< 0.001

Supplementary Table 5: Summary table from binomial generalized mixed model (logit-link) for analysis of parasite prevalence: PV ~ location + (1|river); PV is the proportion of *Tb*-positive samples and the total sample size for each river was used as weights in the model. Model run on a subset of data, only including *Tb* present rivers.

Random effects

Groups		Variance	SD
River	(Intercept)	32.67	5.72

N (obs.): 18
N (River): 9

Fixed effects

	Estimate	SE	*z*	*P*
(Intercept)	6.55	2.65	2.47	0.013
Location (upstream)	-4.97	0.77	-6.49	< 0.001

Supplementary Table 6: Summary table from linear mixed model for analysis of parasite load: sqrt(PL) ~ location + (1|river). Model run on all data.

Random effects

Groups		Variance	SD
River	(Intercept)	5190	72.04
Residual		2749	52.43

N (obs.): 444

N (River): 13

Fixed effects

	Estimate	SE	df	*t*	*P*
(Intercept)	82.32	20.31	12.41	4.05	0.002
Location (upstream)	-47.15	5.07	430.60	-9.30	<2e-16

Supplementary Table 7: Summary table from linear mixed model for analysis of parasite load: pl ~ location + (1|river). Model run on a subset of data, only including *Tb* present rivers.

Random effects

Groups		Variance	SD
River	(Intercept)	5731	75.71
Residual		3571	59.76

N (obs.): 315

N (River): 9

Fixed effects

	Estimate	SE	df	*t*	*P*
(Intercept)	115.03	25.68	8.25	4.48	0.002
Location (upstream)	-65.44	6,82	305.26	-9.60	<2e-16

Supplementary Table 8: Summary table from linear mixed model for analysis of renal hyperplasia (kidney height, K relative to dorsal musculature height, B): K ~ B + location + (1|river). Model is run on all data without separation of infected and *Tb* absent rivers.

Random effects

Groups		Variance	SD
River	(Intercept)	0.21	0.46
Residual		0.19	0.44

N (obs.): 443
N (River): 13

Fixed effects

	Estimate	SE	df	*t*	*P*
(Intercept)	1.12	0.17	38.8	6.4	1.63e-7
Body height	0.04	0.02	440.0	2.2	0.028
Location (upstream)	-0.50	0.04	429.0	-11.8	<2e-16

Supplementary Table 9: Summary table from linear mixed model for analysis of renal hyperplasia (kidney height, K relative to dorsal musculature height, B): K ~ B + location × par.pres + (1|river). Model includes factor parasite presence (par.pres) in interaction with location to compare infected and *Tb* present/absent rivers.

Random effects

Groups		Variance	SD
River	(Intercept)	0.15	0.39
Residual		0.18	0.43

N (obs.): 443
N (River): 13

Fixed effects

	Estimate	SE	df	*t*	*P*
(Intercept)	0.38	0.24	22.5	1.59	0.125
Body height	0.06	0.02	435.1	3.65	0.0003
Location (upstream)	-0.14	0.08	430.5	-1.70	0.090
Parasite presence (infected river)	0.79	0.24	11.9	3.21	0.008
Location (upstream) × Par. pres. (infected river)	-0.51	0.09	431.1	-5.34	1.51e-7

Supplementary Table 10: Estimated qPCR parameters associated with *Tb* quantification. *Calculations were performed with first four ten-fold serial dilution concentrations.

**Parameter**	
Mean SD for technical replicate Cq values	0.110
Median SD for technical replicate Cq values	0.053
25 percentile for technical replicate Cq values	0.031
75 percentile for technical replicate Cq values	0.113
Plate 1 amplification efficiency %	91.45*
Plate 2 amplification efficiency %	92.16
Plate 3 amplification efficiency %	92.25*
Plate 4 amplification efficiency %	93.20
Plate 5 amplification efficiency %	91.68*
Plate 6 amplification efficiency %	93.60
Plate 1 r^2 of calibration curve	0.999
Plate 2 r^2 of calibration curve	0.993
Plate 3 r^2 of calibration curve	0.999
Plate 4 r^2 of calibration curve	0.997
Plate 5 r^2 of calibration curve	0.999
Plate 6 r^2 of calibration curve	0.998
Synthetic dilution series plate efficiency %	91.78
Synthetic dilution series r^2	0.998
