# Supplementary material for: Dams threaten salmonids by triggering temperature-dependent proliferative kidney disease
Source: Commun Biol. 2026 Jan 9;9:192. doi: 10.1038/s42003-025-09470-1 (PMC12881449; doi:10.1038/s42003-025-09470-1)
Supplement: Supplementary file 3 — Reporting Summary [file 42003_2025_9470_MOESM3_ESM.pdf]

nature portfolio

Corresponding author(s): Prof. Anti Vasemägi

Last updated by author(s): Jul 14, 2025

Reporting Summary

Nature Portfolio wishes to improve the reproducibility of the work that we publish. This form provides structure for consistency and transparency in reporting. For further information on Nature Portfolio policies, see our Editorial Policies and the Editorial Policy Checklist.

Statistics

For all statistical analyses, confirm that the following items are present in the figure legend, table legend, main text, or Methods section.

n/a	Confirmed	
☐	☒	The exact sample size (*n*) for each experimental group/condition, given as a discrete number and unit of measurement
☐	☒	A statement on whether measurements were taken from distinct samples or whether the same sample was measured repeatedly
☐	☒	The statistical test(s) used AND whether they are one- or two-sided<br>*Only common tests should be described solely by name; describe more complex techniques in the Methods section.*
☐	☒	A description of all covariates tested
☐	☒	A description of any assumptions or corrections, such as tests of normality and adjustment for multiple comparisons
☐	☒	A full description of the statistical parameters including central tendency (e.g. means) or other basic estimates (e.g. regression coefficient) AND variation (e.g. standard deviation) or associated estimates of uncertainty (e.g. confidence intervals)
☐	☒	For null hypothesis testing, the test statistic (e.g. *F*, *t*, *r*) with confidence intervals, effect sizes, degrees of freedom and *P* value noted<br>*Give P values as exact values whenever suitable.*
☒	☐	For Bayesian analysis, information on the choice of priors and Markov chain Monte Carlo settings
☐	☒	For hierarchical and complex designs, identification of the appropriate level for tests and full reporting of outcomes
☐	☒	Estimates of effect sizes (e.g. Cohen's *d*, Pearson's *r*), indicating how they were calculated

Our web collection on statistics for biologists contains articles on many of the points above.

Software and code

Policy information about availability of computer code

Data collection	The kidney-to-body thickness ratio (K/B-ratio) was measured as a quantitative estimate of kidney hyperplasia using open source image processing tool ImageJ.
Data analysis	All analyses were run in R 4.4.2 (R Core Team, Vienna; https://www.r-project.org).

For manuscripts utilizing custom algorithms or software that are central to the research but not yet described in published literature, software must be made available to editors and reviewers. We strongly encourage code deposition in a community repository (e.g. GitHub). See the Nature Portfolio guidelines for submitting code & software for further information.

Data

Policy information about availability of data

All manuscripts must include a data availability statement. This statement should provide the following information, where applicable:

- Accession codes, unique identifiers, or web links for publicly available datasets
- A description of any restrictions on data availability
- For clinical datasets or third party data, please ensure that the statement adheres to our policy

All data and associated code used within the study is deposited in Figshare https://doi.org/10.6084/m9.figshare.29268938

Research involving human participants, their data, or biological material

Policy information about studies with human participants or human data. See also policy information about sex, gender (identity/presentation), and sexual orientation and race, ethnicity and racism.

Reporting on sex and gender	n/a
Reporting on race, ethnicity, or other socially relevant groupings	n/a
Population characteristics	n/a
Recruitment	n/a
Ethics oversight	n/a

Note that full information on the approval of the study protocol must also be provided in the manuscript.

Field-specific reporting

Please select the one below that is the best fit for your research. If you are not sure, read the appropriate sections before making your selection.

☐ Life sciences ☐ Behavioural & social sciences ☒ Ecological, evolutionary & environmental sciences

For a reference copy of the document with all sections, see nature.com/documents/nr-reporting-summary-flat.pdf

Ecological, evolutionary & environmental sciences study design

All studies must disclose on these points even when the disclosure is negative.

Study description	To investigate the impact of small dams on parasite infection dynamics and host pathology in wild brown trout, we employed a paired upstream - downstream sampling design. This approach allowed us to directly compare environmental and biological parameters across dam-influenced and non-impacted sites while minimizing confounding spatial effects. We selected 14 river systems in Estonia and identified the nearest suitable upstream and downstream habitats for spawning and young-of-the-year (YOY) brown trout. These paired sites were used to assess infection prevalence, parasite load and disease symptoms associated with Tb, the causative agent of PKD. Summer water temperature profiles were recorded using high-resolution loggers (Onset HOBO MX; HOBO Data Loggers, Bourne, MA, USA). Biological sampling and molecular diagnostics were then employed to link dam-induced thermal changes with parasite load and disease symptoms.
Research sample	Standard electrofishing of YOY brown trout was conducted over 12 day period in Aug-Sept 2022 (14 reservoirs, 28 sites). In September 2023, we sampled one additional river. Previous studies have shown that Tb prevalence in infected salmonid populations is typically > 0.2. Therefore, we aimed to collect kidney tissue samples from ca 20 YOY brown trout per location to minimize the number of lethally sampled fish, while maintaining sufficient power (95%) to distinguish non-infected and infected populations when true prevalence is > 0.2.
Sampling strategy	Previous studies have shown that Tb prevalence in infected salmonid populations is typically > 0.2. Therefore, we aimed to collect kidney tissue samples from ca 20 YOY brown trout per location to minimize the number of lethally sampled fish, while maintaining sufficient power (95%) to distinguish non-infected and infected populations when true prevalence is > 0.2.
Data collection	Fork length and total mass (mg) of each fish was measured with a precision scale (Radwag WLC 2/A2/C/2; RADWAG, Radom, Poland). Blood samples were collected from the caudal artery using heparinized microcapillary tubes (0.5–0.6 mm in diameter; Paul Marienfeld GmbH & Co.KG, Lauda-Königshofen, Germany) and centrifuged immediately after sampling at 12,250 g for five minutes (QBC Capillary Centrifuge; Drucker Diagnostics, Port Matilda, PA, USA). Blood plasma and packed red blood cell estimates were carried out using a standard ruler (to closest 0.5 mm). One, two or three microcapillaries were collected per fish from 427, 222 and 6 individuals, respectively, and mean hematocrit values per fish was calculated when multiple estimates were available. In order to quantify renal hyperplasia, a sagittal cross section of each fish was produced by cutting the body between the front tip and the end of the dorsal fin (37). This section was photographed against 1 mm grid (Fig. 1J) with a digital camera for the measurement of the kidney-to-body thickness ratio (K/B-ratio) as a quantitative estimate of kidney hyperplasia using ImageJ software (37, 66). The cross section was stored in 96% ethanol (5 mL screw-cap centrifuge tubes) for DNA extraction from the kidney tissue for quantification of T. bryosalmonae (Tb). Total genomic DNA from kidney tissues of the 446 YOY brown trout were extracted using QIAamp 96 DNA QIAcube HT kit and the QIAcube® HT Instrument for automated nucleic acid purification (QIAGEN, Hilden, Germany). DNA concentrations were measured with NanoDrop 2000 (Thermo Fisher Scientific, Waltham, MA, USA) and diluted to 20 ng µL-1. The quantification of Tb was performed in a set of three replicates for each kidney extraction using real-time quantitative PCR (qPCR) based on Taqman probe on a LightCycler 480 (Roche, Basel, Switzerland). Water temperature data was measured every six hours (06:00, 12:00, 18:00, and 00:00). For all studied locations the mean summer water temperature was calculated for the period of June 29 to August 22 (55 days; longer time series available for 13 rivers). Based on maximum and minimum daily temperatures, we calculated diurnal variation for the same period of time (Table S1, Fig. S1). Dam height and reservoir area data was obtained from the Estonian Environmental Portal public database (https://register.keskkonnaportaal.ee/).

Timing and spatial scale	Standard electrofishing of YOY brown trout including in situ data collection was conducted over 12 day period in Aug-Sept 2022 (14 reservoirs, 28 sites). DNA extractions and molecular quantification of the parasite was conducted from January to May of 2023. In September 2023, we sampled one additional river. DNA extractions and molecular quantification of these samples were conducted in Autumn 2023.
Data exclusions	No data was excluded from the analysis.
Reproducibility	The quantification of Tb from kidney tissue was performed in a set of three replicates for each kidney extraction using real-time quantitative PCR (qPCR) based on Taqman probe on a LightCycler 480 (Roche, Basel, Switzerland). All qPCR plates were manually prepared and run by the same individual. Due to the substantial variation in parasite load among infected samples (e.g., up to 1.6×10^6 orders of magnitude), an SD threshold of <1 was used to identify outliers (mean SD 0.110, median SD 0.053). Parasite load was also quantified from spleen tissue of the same individuals, showing strong correlation between the two tissues, demonstrating reliability of the analyses (data shown in https://doi.org/10.1111/jfd.14148).
Randomization	Quantification of Tb was performed on six 384 qPCR plates. Samples were allocated throughout the six plates in a site- and population based sequence, every plate had samples from both upstream and downstream sites.
Blinding	To reduce potential bias, kidney tissue samples were processed in a randomized order with respect to site (upstream/downstream) and river population. While the researcher performing DNA extractions and qPCR analyses was aware of the sample order, the specific origin of each sample (i.e., site and population) was blinded during laboratory procedures.

Did the study involve field work? ☒ Yes ☐ No

Field work, collection and transport

Field conditions	Fieldwork was carried out during a dry period characterized by mild summer temperatures, averaging approximately 20 °C.
Location	We selected 14 river systems in Estonia and identified the nearest suitable upstream and downstream habitats for spawning and young-of-the-year (YOY) brown trout from the dams. The study sites comprised shallow river stretches with water depths up to 50 cm.
Access & import/export	Standard electrofishing of young-of-the-year (YOY) brown trout was conducted under permit no. 10-1/22/42-2 for 2022 and permit no. 10-1/23/50-2 for 2023, issued by Ministry of Regional Affairs and Agriculture of Estonia.
Disturbance	To minimize potential disturbance caused by electrofishing, the procedure was conducted following established ethical guidelines and standardized protocols, using the lowest effective voltage and limiting sampling duration and area. Fish were handled carefully and specimens not included in the study were released immediately to reduce stress and avoid prolonged disruption to the habitat.

Reporting for specific materials, systems and methods

We require information from authors about some types of materials, experimental systems and methods used in many studies. Here, indicate whether each material, system or method listed is relevant to your study. If you are not sure if a list item applies to your research, read the appropriate section before selecting a response.

Materials & experimental systems

n/a	Involved in the study	
☒	☐	Antibodies
☒	☐	Eukaryotic cell lines
☒	☐	Palaeontology and archaeology
☐	☒	Animals and other organisms
☒	☐	Clinical data
☒	☐	Dual use research of concern
☒	☐	Plants

Methods

n/a	Involved in the study	
☒	☐	ChIP-seq
☒	☐	Flow cytometry
☒	☐	MRI-based neuroimaging

Animals and other research organisms

Policy information about studies involving animals; ARRIVE guidelines recommended for reporting animal research, and Sex and Gender in Research

Laboratory animals	The study did not involve laboratory animals.
Wild animals	Standard electrofishing of young-of-the-year (YOY) brown trout was conducted (14 reservoirs, 28 sites, permit no. 10-1/22/42-2 for 2022 and permit no. 10-1/23/50-2 for 2023, issued by Ministry of Regional Affairs and Agriculture of Estonia). Caught YOY brown

	trout were killed with an overdose of benzocaine (> 250 mg L-1, Caesar & Loretz GmbH, Hilden, Germany). Previous studies have shown that Tb prevalence in infected salmonid populations is typically > 0.2. Therefore, we aimed to collect tissue samples from ca 20 YOY brown trout per location to minimize the number of lethally sampled fish, while maintaining sufficient power (95%) to distinguish non-infected and infected populations when true prevalence is > 0.2.
Reporting on sex	The sex of the young-of-the-year (YOY) fish was not determined due to the difficulty of visually distinguishing sexual characteristics at such an early developmental stage.
Field-collected samples	Kidney tissue samples of the YOY brown trout were stored in 96% ethanol (5 mL screw-cap centrifuge tubes) for DNA extraction from the kidney tissue for quantification of T. bryosalmonae (Tb). The samples were kept in -20 °C until DNA extractions. Following extraction, the remaining tissue samples were retained at −20°C as a precautionary measure.
Ethics oversight	Fish sampled in the study were euthanised in accordance with the principles described in Directive 2010/63/EU of the European Parliament and of the Council of 22 September 2010 on the protection of animals used for scientific purposes.

Note that full information on the approval of the study protocol must also be provided in the manuscript.

Plants

Seed stocks	n/a
Novel plant genotypes	n/a
Authentication	n/a
